# Quantifying Eye‐Tracking‐Based Behavioral Strategy Transitions Using Markov Models: A Novel Indicator of Cognitive Control Impairments in Mild Cognitive Impairment

**DOI:** 10.1002/alz70861_108021

**Published:** 2025-12-23

**Authors:** Kahye Kim, Joong Il Kim, Jiwoo Suk, Jin Mi Chun, Jaeuk Kim

**Affiliations:** ^1^ Korea Institute of Oriental Medicine, Daejeon, Daejeon Korea, Republic of (South); ^2^ Korea Institute of Oriental Medicine, Daejeon Korea, Republic of (South); ^3^ Korea Institute of Oriental Medicine, Daejeon, Yuseong Gu Korea, Republic of (South)

## Abstract

**Background:**

Mild cognitive impairment (MCI), often regarded as a prodromal stage of Alzheimer’s disease (AD), is associated with early deficits in cognitive control and behavioral regulation. This study aimed to develop quantitative behavioral biomarkers for early‐stage detection by modeling response dynamics in a structured visuomotor task.

**Method:**

From the GARD (Gwangju Alzheimer's and Related Dementias) Cohort, data were analyzed from 120 older adults (60 cognitively normal and 60 with mild cognitive impairment) who had completed an anti‐saccade eye‐tracking task. Each response was classified into one of seven categories: Correct, Uncorrected‐Inhibition, Self‐Corrected, Anticipatory, Omission, Invalid, or Miss Correct. First‐order Markov transition probability matrices were computed for each participant. Five strategy‐based indices were derived from these matrices: Accuracy Stability Index (ASI), Error‐Driven Correction Index (EDCI), Inhibition Failure Index (IFI), Self‐Regulation Index (SRI), and Attention Loss Index (ALI).

**Result:**

Participants with MCI showed significantly lower ASI and EDCI and higher IFI scores compared to cognitively normal controls (p < .05). ASI demonstrated a moderate positive correlation with frontal executive function scores (r ≈ 0.5). In multivariate logistic regression adjusted for age, sex, and education, both ASI (B = ‐1.449, 95% CI: 0.065–0.844) and EDCI (B = ‐1.367, 95% CI: 0.077–0.847) significantly predicted MCI status.

**Conclusion:**

Markov‐based transition metrics capture dynamic impairments in cognitive regulation that are not reflected in conventional error rates. Given the transitional nature of MCI toward AD, these indices may aid in identifying individuals at risk for progression. Further validation through longitudinal studies is warranted.

